# From Treatment to Tragedy: Severe Methotrexate Toxicity With Mucocutaneous Ulcers, Myelosuppression, and Nephropathy

**DOI:** 10.7759/cureus.57797

**Published:** 2024-04-08

**Authors:** Rucha Sawant, Pranav Chaudhari, Nidhi A Bardiya, Sourya Acharya, Sunil Kumar

**Affiliations:** 1 Medicine, Jawaharlal Nehru Medical College, Wardha, IND

**Keywords:** pancytopenia, bone marrow depression, mucocutaneous ulceration, chronic kidney disease, methotrexate toxicity

## Abstract

Methotrexate (MTX) is a well-established drug for the use of various neoplastic disorders. Recently, it has been widely used as a disease-modifying antirheumatic drug (DMARD) in low doses, mainly for rheumatoid arthritis (RA) and psoriasis. The drug is known to cause renal damage as well as be excreted via the kidneys, thus causing a higher incidence of adverse effects in patients with impaired renal function. The side effects of MTX toxicity range from mucocutaneous ulcers to nephrotoxicity and bone marrow depression, all of which are seen in this case.

Here, we report an elderly male in his late 60s who was prescribed MTX 15 mg once a week along with folic acid 5 mg for RA by a general practitioner. Despite being prescribed once weekly, he continued to take MTX daily without following up with a physician for a span of five months. Following this, he presented to the medicine outpatient department with odynophagia due to oral ulcers for 10 days. He was diagnosed with MTX toxicity, causing nephropathy, myelosuppression, and mucocutaneous ulcerations. He was treated with injectable leucovorin 100 mg thrice a day until the toxicity subsided, leading to his eventual recovery.

## Introduction

Low-dose methotrexate (LD-MTX) is used as a disease-modifying antirheumatic drug (DMARD) for treating rheumatoid arthritis (RA), inducing remission, and reducing disease activity in these patients. The body eliminates this substance through a two-step process: liver breakdown and kidney removal. Due to the drug's predominant renal excretion, its predisposition to toxicity in patients with renal dysfunction is well-known, and the drug is even contraindicated in patients with advanced chronic kidney disease (CKD) with creatinine clearance less than 30 mL/min [1]. However, the effects in patients with mild to moderate CKD are unknown [1]. The degree of CKD in patients with RA is about 9-24% [2]. Some guidelines suggest decreasing the dose of methotrexate (MTX) according to creatinine clearance [3].

Patients receiving MTX, especially those with underlying renal dysfunction, should be monitored for hepatotoxicity, myelosuppression, and mucositis. Through this report, we wish to emphasize the importance of counseling patients at the time of starting MTX about the drug's toxicities and a thorough evaluation of the patient for any preexisting renal or hepatic impairment before beginning the same, as well as monitoring for drug toxicity during treatment.

## Case presentation

**Figure 1:**
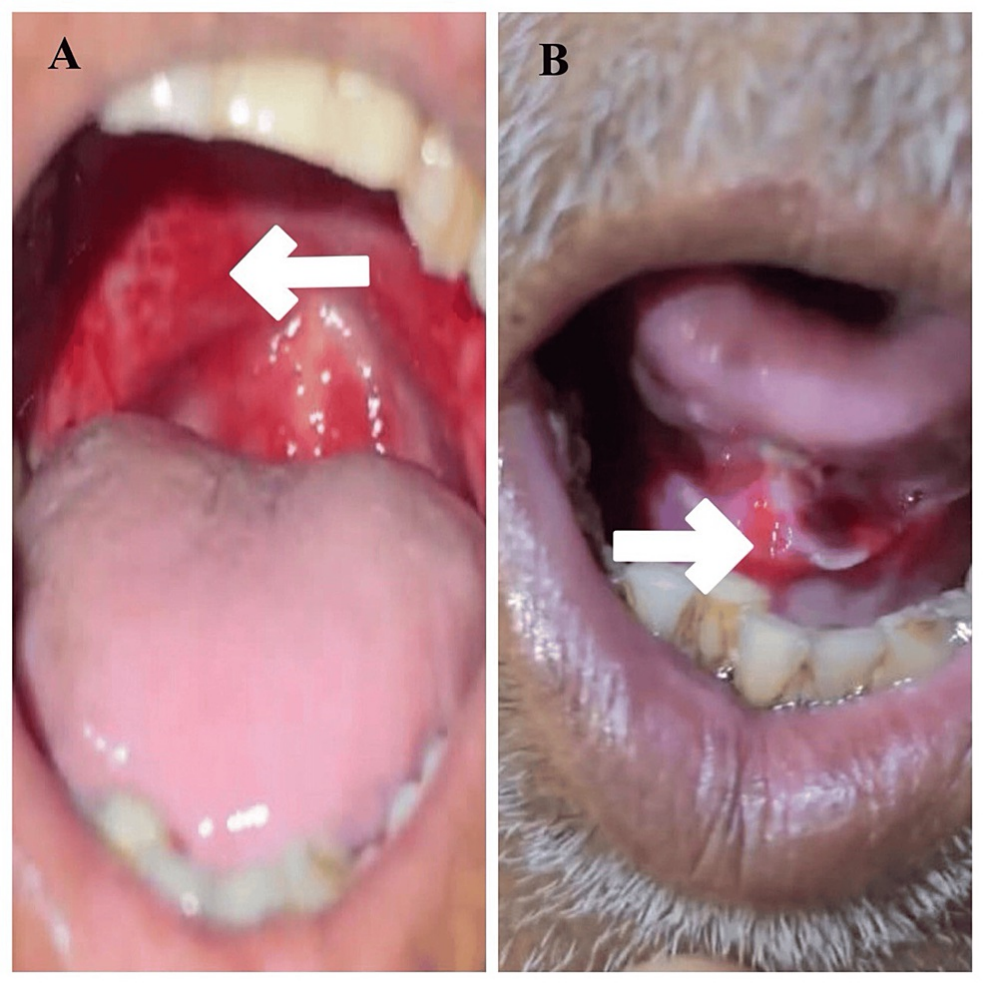
(A) The arrow shows an erythematous ulcer in the posterior pharyngeal wall. (B) The arrow shows an erythematous ulcer in the base of the tongue.

An elderly male in his early 60s presented with complaints of inability to swallow or completely open his mouth due to severe pain in the oral cavity for 10 days. Two months ago, he visited a local general practitioner, where he was diagnosed with RA, for which he was started on a tablet of MTX 15 mg once a week for the last five months, along with folic acid 5 mg once a day. Upon further enquiring and taking a detailed history, it was revealed that the patient was taking MTX 15 mg once daily. The patient’s general examination revealed a heart rate of 72 beats per minute, blood pressure of 130/80 mmHg, respiratory rate of 18 cycles per minute, and an oxygen saturation of 98% on ambient air. He also has pallor. Upon examining his oral cavity, he was found to have an extensive erythematous ulcer in the posterior oral cavity (Figure 1A and Figure 1B).

**Figure 2:**
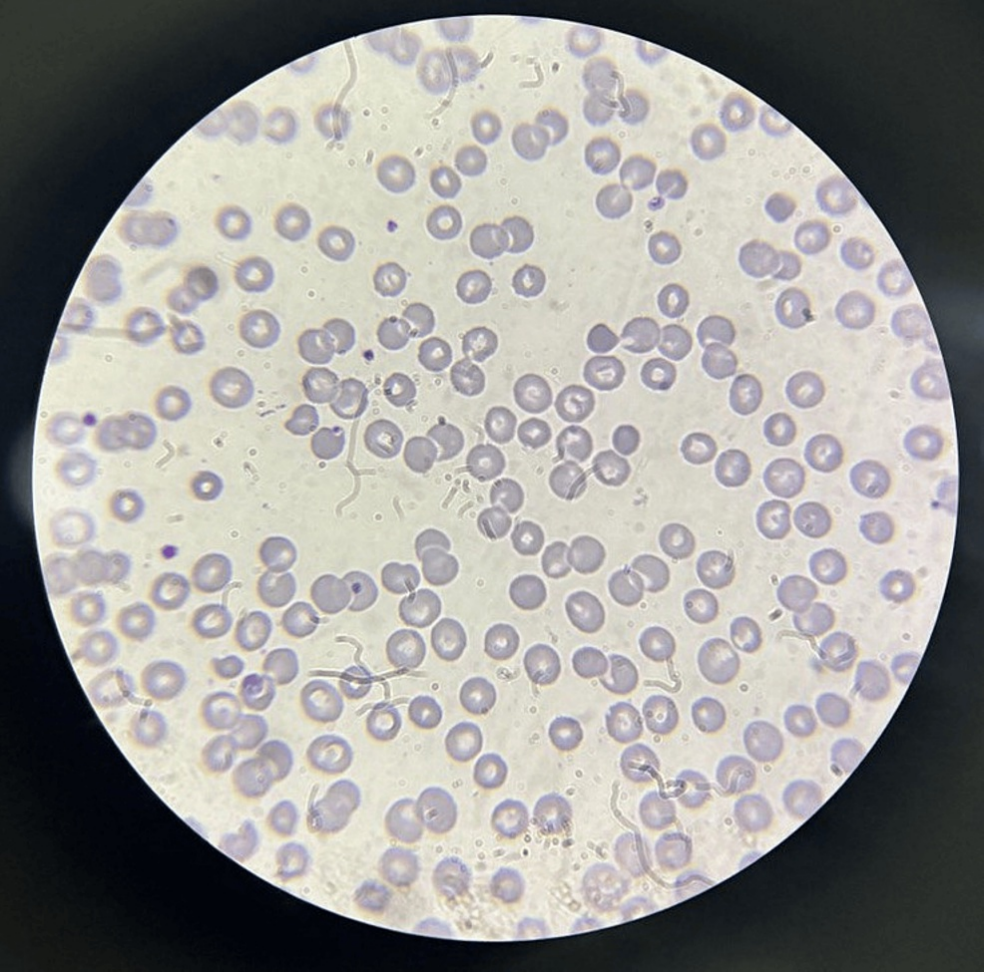
Peripheral smear showing the macro-normocytic normochromic red blood cells with reduced platelet on the smear.

His cardiovascular, abdominal, respiratory, and neurological examination showed no abnormal findings. Upon performing laboratory investigations, he was found to have anemia and thrombocytopenia with a decreased hemoglobin and platelet count and increased serum ferritin. His peripheral smear showed the macro-normocytic normochromic type of anemia with reduced absolute platelet count (Figure 2).

He also had an increased serum creatinine and urea with a reduced creatinine clearance of 10 mL/min, and his estimated glomerular filtration rate (eGFR) was 24 mL/min/1.73 m^2^. His liver function test showed no abnormality. He was suspected of having MTX toxicity, so his serum MTX levels were done, which were raised after 72 hours of the last dose (Table 1).

**Table 1:** Laboratory parameter LDH, lactate dehydrogenase; MCV, mean corpuscular volume; WBC, white blood cell

Laboratory parameter	Patient’s values on admission	Patient’s values on discharge	Reference values
Hemoglobin (g/dL)	9.1	11.1	12-18
MCV	102	89	81-102
WBC (/mm^3^)	3,500	4,600	4,500-11,000
Total platelet (×109/L)	0.32	1.3	1.4-4.5
Urea (mg/dL)	22	20	9-22
Creatinine (mg/dL)	3.4	2.5	0.6-1.25
Sodium (mg/dL)	142	144	137-144
Potassium (mg/dL)	4.8	4.5	3.5-5.2
Magnesium (mg/dL)	1.7	1.8	1.7-2.2
Phosphate (mg/dL)	4.1	4.5	2.5-4.4
Calcium (mg/dL)	7.1	7.4	8.4-10.1
Serum ferritin (ng/mL)	602	-	17.5-464
Serum iron (mcg/dL)	82	-	49-181
Serum LDH (U/L)	150	-	120-246
Serum methotrexate	0.9	0.05	<0.1 µmol/L

On abdominal ultrasound, both his kidneys were shrunken, the right kidney being 6 × 3.4 cm and the left being 6.7 × 3.5 cm, with loss of corticomedullary differentiation. His urinary albumin creatinine ratio was 28 mg/g. Thus, he was diagnosed with CKD stage G4A1. He was advised to stop taking the MTX and was prescribed betamethasone mouthwash. A rheumatologist consultation was taken, and he was started on injectable leucovorin 100 mg thrice a day for five days. His serum MTX levels were repeated and found to be reduced; then, leucovorin was stopped. He was also given parenteral iron supplementation, as iron sucrose 200 mg once daily for five days. At discharge, the patient had a creatinine clearance of 25 mL/min. He was discharged on MTX 7.5 mg once a week on Sunday and folic acid 5 mg six days a week once daily.

## Discussion

Since the 1980s, clinical investigations have shown that MTX effectively manages RA [4]. As a result, its use has increased significantly. The dosage of MTX for treatment varies from 5 to 25 mg/week, with a low starting dose being common [5]. The optimal dose is determined by a balance between effectively managing the disease and minimizing side effects [6].

MTX is used as an initial drug for the treatment of RA and plays a central role in its treatment. It is a DMARD, which remains the cornerstone of treatment even after newer drugs are introduced, owing to its potency and efficacy. It also treats systemic lupus erythematosus, multiple sclerosis, psoriasis, juvenile idiopathic arthritis, and inflammatory bowel disease [6]. Recently, its use has been seen in viral arthritis caused by viruses like parvovirus B19, hepatitis virus, and human immunodeficiency virus [6]. The immunomodulatory effect of MTX is due to several mechanisms, including alteration of the cytokine networks, adenosine signaling modulation, producing reactive oxygen species, and high-mobility group box 1 (HMGB1) alarmin suppression [6]. Oral MTX, a folic acid antagonist, is a prodrug that undergoes a process of polyglutamation inside the cell over 27.5 weeks, explaining why it takes longer to reach a steady therapeutic value [7]. It competes and inhibits the folate-dependent enzymes, thus inhibiting purine and pyrimidine synthesis needed for DNA and RNA formation, which impede the growth of cells. However, the mechanism of LD-MTX still needs to be understood [6]. Most of the drug is excreted by the kidneys unchanged, so any impairment in the glomerular filtration (GFR) results in the accumulation of the drug and slow release over time, resulting in toxicity, mainly bone marrow depression [6].

The adverse effects of MTX toxicity can be divided into two subsets: non-life-threatening ones like headache, fatigue, and mucositis and life-threatening ones like myelosuppression, pneumonitis, and liver cirrhosis [6]. Of these, mucositis, myelosuppression, and nephrotoxicity are seen in patients in this report. The most common are gastrointestinal complications and hepatotoxicity. Folic acid supplementation cannot manage nephrotoxicity and pulmonary fibrosis [6].

Mucocutaneous ulceration, an adverse effect of MTX, is due to a lack of folic acid supplements or an overdose due to confusion about the once-weekly regimen. MTX primarily affects cells with quick turnover, such as mucosa and bone marrow, leading to bone marrow depression and mucocutaneous ulcers [8]. Though dose-dependent, research indicated that up to 55% of individuals who took LD-MTX experienced oral mucositis over 90 months [8].

It is unclear if supplementation with folic acid reduces the incidence of cytopenia [9]. Additionally, folic acid given at regular doses might not be effective once MTX rises due to nephropathy [9]. One research found that hepatotoxicity and pancytopenia occurred at a frequency of 7.5% and 5%, respectively [2]. While another report suggested clinically significant pancytopenia in 2% of RA patients of MTX [10], although hematological abnormalities due to MTX use in RA are rare, pancytopenia has an incidence of 78% of all hematological abnormalities. It even has a high mortality rate [9].

Although the exact mechanism is unknown, LD-MTX causing renal damage is a well-established fact [6]. High-dose MTX causes renal damage by urinary precipitation of a metabolite 7-hydroxy-MTX (7-OH MTX), causing tubular injury. However, this is rarely seen in chronic LD-MTX use [6]. Patients with stage 3 renal failure see a considerable decrease in MTX clearance. However, individual patient pharmacokinetics vary greatly, making prediction impossible [11]. For this reason, LD-MTX is contraindicated in the GFR less than 30 mL/min [6]. The patient reported an eGFR of 24 mL/min/1.73 m^2^.

Treatment modalities include folinic acid supplementation, appropriate antibiotics, packed red cell transfusion, hemodialysis, platelet transfusion, and granulocyte colony-stimulating factor [11]. Parallel use of hydroxychloroquine could guard against MTX-related toxicity in patients of RA who have impaired renal function [2]. Glucarpidase is authorized to treat MTX toxicity when it's above one μmol/L in patients of RA who have nephropathy [4]. Patients are advised to follow up and monitor laboratory investigations every three months after six months of starting MTX [12].

The clinical response to MTX varies significantly among individuals, both in terms of effectiveness and toxicity, with responses ranging from 50-70% [6]. Individual patient characteristics (age, sex, comorbidities) and hereditary factors contribute to the diversity in MTX effects across different patients.

## Conclusions

LD-MTX is used to improve the condition of patients in many inflammatory autoimmune disorders, although toxicity remains a concern. Despite its usefulness in treating disorders such as RA and psoriasis, misusing MTX, especially in older patients, can result in significant adverse effects, including liver cirrhosis, lung fibrosis, nephropathy, and hematological abnormalities. Knowledge of MTX's fundamental mechanisms of action offers more insights into MTX-related toxicity. Patients require comprehensive counseling on the risks associated with MTX treatment. Additionally, they should be advised to maintain frequent follow-up appointments while on MTX to enable early detection of any potential toxicities, allowing for timely treatment.
